# An Unexpected Transient Breakdown of the Blood Brain Barrier Triggers Passage of Large Intravenously Administered Nanoparticles

**DOI:** 10.1038/srep22595

**Published:** 2016-03-04

**Authors:** Nicole M. Smith, Ivana Gachulincova, Diwei Ho, Charlotte Bailey, Carole A. Bartlett, Marck Norret, John Murphy, Alysia Buckley, Paul J. Rigby, Michael J. House, Timothy St. Pierre, Melinda Fitzgerald, K. Swaminathan Iyer, Sarah A. Dunlop

**Affiliations:** 1 Experimental and Regenerative Neurosciences, School of Animal Biology, The University of Western Australia, Perth, WA 6009, Australia; 2 School of Chemistry and Biochemistry, The University of Western Australia, Perth, WA 6009, Australia; 3 Centre for Microscopy, Characterisation and Analysis, The University of Western Australia, Perth, WA 6009, Australia; 4 School of Physics, The University of Western Australia, Perth, WA 6009, Australia

## Abstract

The highly restrictive blood-brain barrier (BBB) plays a critically important role in maintaining brain homeostasis and is pivotal for proper neuronal function. The BBB is currently considered the main limiting factor restricting the passage of large (up to 200 nm) intravenously administered nanoparticles to the brain. Breakdown of the barrier occurs as a consequence of cerebrovascular diseases and traumatic brain injury. In this article, we report that remote injuries in the CNS are also associated with BBB dysfunction. In particular, we show that a focal partial transection of the optic nerve triggers a previously unknown transient opening of the mammalian BBB that occurs in the visual centres. Importantly, we demonstrate that this transient BBB breakdown results in a dramatic change in the biodistribution of intravenously administered large polymeric nanoparticles which were previously deemed as BBB-impermeable.

The blood brain barrier (BBB) plays a critical role in controlling the brain microenvironment, restricting the passage of intravenously administered nanoparticles^1^. Nanoparticles that can breach this highly restrictive barrier depend on size (as large as 114 nm after dense PEG surface modification)^2^ and surface functionalizations that enable receptor mediated endocytosis and transcytosis^3^,^4^,^5^,^6^,^7^. The current knowledge of BBB breakdown is associated with a direct traumatic injury to the brain or with cerebrovascular diseases such as stroke, which can result in unregulated passage across the barrier. However, the status of the BBB as a consequence of a remote injury in the central nervous system (CNS) is little understood. Understanding this is pivotal in our examination of the safety of intravenously administered carriers as unexpected BBB dysfunctions can alter the biodistribution of large nanoparticles that have been previously deemed as BBB-impermeable. Endogenous plasma protein albumin functionalised nanoparticles have emerged as one of the most important non-immunogenic BBB impermeable intravenous carriers for treatment of diseases like cancer^8^,^9^. Herein, we demonstrate that a focal partial transection of the optic nerve, which is distinct from the brain, triggers a previously unknown transient opening of the BBB in the brain resulting in a dramatic change in the biodistribution of systemically administered large (up to 200 nm) albumin-functionalised polymeric nanoparticles and consequently their accumulation in the brain.

Brain injury, whether ischemic, hemorrhagic or traumatic, leads to dysfunction of the BBB. Additionally, BBB dysfunction is a hallmark of several neurological conditions including multiple sclerosis and stroke^10^. BBB disruption is associated with concomitant spread of vasogenic brain oedema and axonal injury from a primary brain lesion to areas neuroanatomically connected to the injured region^11^,^12^. Moreover, both traumatic and inflammatory brain injuries are directly associated with increased inter- and intracellular transport making the BBB tight junctions leaky^13^. This BBB impairment results in cross talk between the immune and nervous systems resulting in long-term molecular and cellular changes that significantly hamper recovery^14^,^15^. Trauma to the optic nerve, an “outpost” of the brain, is also associated with both acute damage induced by the primary injury and progressive secondary degeneration of intact, but highly vulnerable tissue^16^,^17^,^18^. This progressive degeneration in the mammalian CNS is associated with an inability to regenerate and an inhibitory milieu. Interestingly in the case of frogs, optic nerve regeneration is accompanied by a transient opening of the BBB whereas injury without regeneration following optic nerve ligation does not lead to BBB breakdown^19^. However, in mammals, little is known of the BBB status during the spread of secondary degeneration away from the initial injury site in the CNS such as the optic nerve. In the case of the injured mammalian CNS, early intervention is critical to prevent progression of secondary degeneration and consequently enable preservation of function. It is therefore essential to determine BBB status, to enable identification of CNS regions requiring intervention in the early phase of injury to prevent progression of secondary degeneration.

In the present study we used our extensively characterized partial optic nerve transection model in adult (160–180 g, ~3 months) piebald viral Glaxo (PVG) female hooded rats to answer the aforementioned question^17^,^18^. The optic nerves from the two eyes are distinct from the brain and intersect at the optic chiasm, where most axons decussate to form the optic tracts in the brain. The merit of the partial optic nerve transection model is that the strict topographic arrangement of retinal ganglion cells and their axons at the injury site in the PVG rat strain enables accurate separation of the primary injury site from secondary degeneration in the remaining tissue^18^. Indeed, this model has allowed extensive characterization of secondary degeneration in tissue local to the injury (i.e. the ipsilateral (injured) optic nerve and retina^17^,^18^) but regions remote to the injury site (i.e. in the brain), including BBB breakdown have yet to be investigated. It is important to note that the partial optic nerve transection model additionally enables analysis of the distinct morphological and molecular changes associated with BBB dysfunction immediately following the injury, before the onset of Wallerian degeneration. While it is known that Wallerian degeneration of transected distal axons will lead to increased inflammatory cell infiltration and may impact upon BBB integrity, Wallerian degeneration is slow in the mammalian CNS, taking months to years^20^. This is believed to be associated with the lack of extensive opening of the BBB following injury, which prevents the entrance of serum opsonins and peripheral macrophages in distal white matter tracks, and results in the persistence of myelin debris in the CNS contributing to a non-regenerative environment^20^. As such, any impact of injury to the optic nerve on remote BBB integrity in the brain is unlikely to be caused by Wallerian degeneration associated inflammation if assessed in the early days following injury. It is also important to note that while BBB breakdown following a focal ischemic injury in the brain in a neurodegenerative model of stroke has been demonstrated to be an age dependent phenomenon^21^. In the absence of a neurodegenerative condition a well-developed tight BBB is formed prior to parturition^22^,^23^. It has been shown that the cellular and paracellular permeability to ions, of vessels in a rat of 21-days gestation is no different from rats 33 days after birth^23^. In the present study, we built on our partial optic nerve transection model in adult female PVG hooded rats, to characterize the nature of the BBB dysfunction following a remote injury in the CNS.

## Results and Discussion

**Figure 1:**
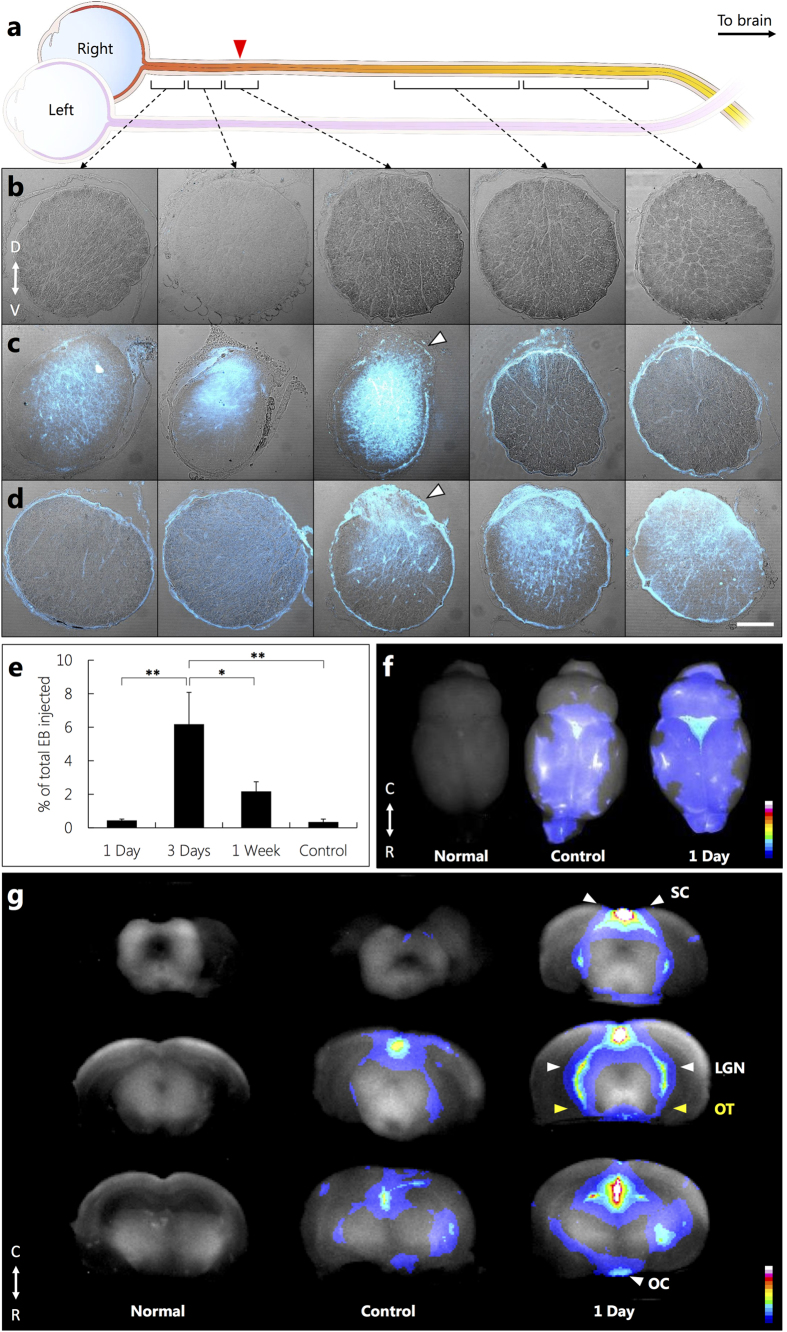
Tracking Evans Blue (EB) fluorescence to establish a compromised BBB following partial optic nerve transection. **(a)** Schematic of sampled right (injured) optic nerve regions with injury site indicated by red arrow. **(b–d)** Transmitted light images with overlaid confocal EB fluorescence images of transverse sections of the right optic nerve of (**b**) control following EB administration, (**c,d**) injured following EB administration at **(c)** 1 day and **(d)** 3 days after injury. Injury site depicted using white arrow in (**c**) and (**d**). D and V represent dorsal and ventral side of the optic nerve, respectively. Sections are positioned from left to right, based on proximity to eye (left) and the brain (right). (**b–d)** Scale bar 100 μm. **(e**) Spectrofluorimetric analysis of extracted EB from the brain of 1 day, 3 days, 1 week and control rats. The results representing the specific absorbance of EB at 620 nm were normalised using the amount of EB detected in the blood stream and expressed as percentage of the amount of EB injected. Data are presented as average ± S.E. (*n* = 5 per group, *P < 0.05, **P < 0.01, one-way ANOVA with Tukey’s multiple comparisons test). (**f**) Multispectral images of the whole brain: (left, Normal) uninjured with no EB administration; (middle, control) uninjured with EB tail vein injection; (right, 1 day) injured with EB tail vein injection 1 day after injury. **(g)** Multispectral heat maps of EB fluorescence intensity obtained from the coronal sections of the brains in (**f)**: (left, Normal); (middle, Control); (right, 1 day). Intense EB signal (1 day) observed along the optic chiasm (OC), the optic tracts (OT), lateral geniculate nucleus (LGN) and superior colliculus (SC), in the brain. C and R represent caudal and rostral side of the brain, respectively; colour code represents degree of EB vascular leakage, where white/red is high and dark blue is low in **f** and **g**.

To assess barrier permeability following injury, adult PVG rats were divided into 1 day, 3 days and 7 days (1 week) post-injury and control groups,. Thus days correspond to the number of days after partial optic nerve transection in adults whereupon a tail vein injection of Evans Blue (EB) was administered. The control group consisted of uninjured adult rats with EB administration. It is noteworthy that EB (MW, 961 Da) is a well-established dye used for quantitative assessment of the BBB permeability by tracking plasma albumin. It is a tetrasodium diazo salt which irreversibly binds to plasma albumin in a 10:1 molar ratio forming ahigh-molecular weight complex (EB-albumin, 68,500 Da) with limited penetration in an uncompromised BBB^24^. In all cases, EB was allowed to circulate for 1 hour after which rats were perfused with 0.9% saline to flush out the vascular system prior to harvesting optic nerves and brains. Barrier permeability was assessed by whole brain multispectral imaging, high-resolution confocal imaging and spectrofluorimetric analysis of EB fluorescence. Confocal microscopy of optic nerve sections indicated intense EB fluorescence at the site of partial optic nerve transection at 1 day post injury and most notably a progression of EB fluorescence along the length of the injured optic nerve towards the brain at 3 days post injury (Fig. 1c,d). This coincides well with our previous findings that reported the inflammatory response was maximal at 3 days after injury along the length of the injured optic nerve^17^. Importantly EB fluorescence was absent in the contralateral (uninjured) optic nerve despite 1–2% decussation of the optic nerve axons at the optic chiasm (Supplementary Fig. 1b–d). Additionally, no EB fluorescence was evident in the optic nerves of controls (Fig. 1b). To evaluate whether progression of EB fluorescence along the optic nerve towards the brain resulted in a concomitant change in BBB permeability, we performed spectrofluorimetric analysis of EB extracted from homogenized rat brains. A statistically significant increase in EB concentration was measured at 3 days following partial optic nerve transection and this was followed by a decrease at 1 week, with levels that were not statistically different from control (Fig. 1e).

Although spectrofluorimetric analysis established that a partial optic nerve transection in adult rats triggers a transient opening of the BBB in the brain, the technique is limited by sensitivity to detect low concentrations of EB during the early phase of BBB dysfunction (1 day). Indeed, accurate early evaluation of BBB compromise is pivotal as it is now widely accepted that following neurotrauma, replacing tissue lost to the primary injury is a major challenge, however, limiting the progression of secondary degeneration in the early phase is a more realistic goal. To examine the early phase of BBB compromise, we analyzed the brain at 1 day after partial optic nerve transection using multispectral imaging of whole brains. EB fluorescence was evident in visual regions of the brain at 1 day after partial optic nerve transection namely around the optic chiasm, the optic tract to the lateral geniculate nucleus and the superior colliculus (Fig. 1f,g and Supplementary Fig. 2). It should be noted that there is a very low EB fluorescence signal in the multispectral images of the control brain, which is attributed to regions of the brain with no BBB such as the choroid plexus and circum-ventricular organs (Fig. 1f,g and Supplementary Fig. 2)^25^.

**Figure 2:**
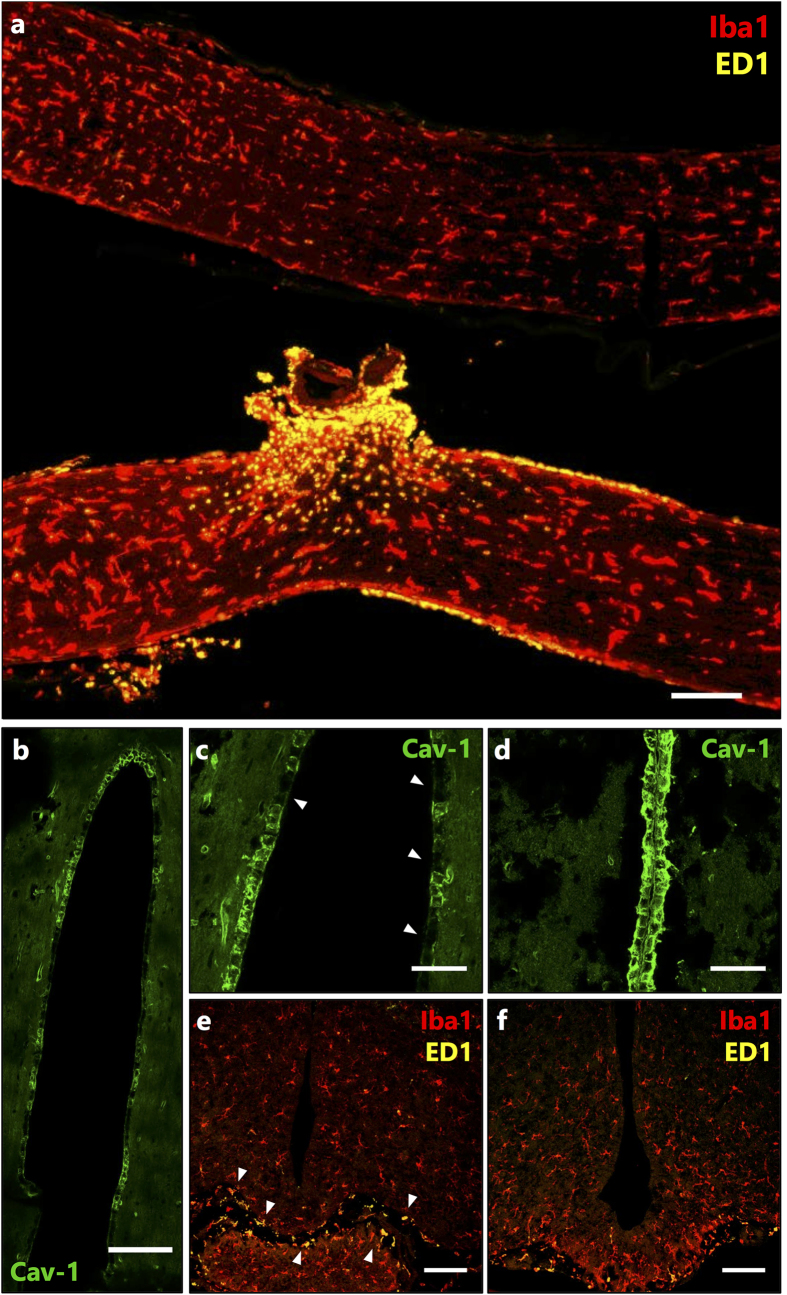
Immunohistochemical evaluation of the optic nerve and the brain using confocal imaging following partial optic nerve transection. (**a**) Iba1 (red) stained all (activated and resting) microglia and macrophages while ED1 (yellow) stained activated microglia and macrophages in the contralateral (uninjured, top) and ipsilateral (injured, bottom) optic nerve 1 day following injury. Scale bar: 500 μm. (**b–d**) Changes in the tight junction protein Caveolin-1 (Cav-1) expression at the 3^rd^ ventricle of the brain. (**b,c)** are representative images of the ventricle (where c is a zoom in of (**b**) showing disruption in Cav-1 staining (indicated by white arrows) 1 day post partial optic nerve transection and (**d**) representative image of continuous Cav-1 staining around perimeter of ventricle in the absence of partial optic nerve transection (control). Scale bar (**b**): 100 μm, (**c**) & (**d**): 50 μm. (**e,f**) Iba1 (red) stained activated and resting microglia and macrophages and ED1 (yellow) stained activated microglia and macrophages at the 3rd ventricle of the rat brain in (**e**) injured (1 day) (indicated by white arrows) and (**f**) uninjured (control). Scale bar: 100 μm.

The propagation of secondary degeneration from the primary injury site is associated with an influx of inflammatory mediators, recruitment of microglia/macrophages and activation of astrocytes^26^. It has been demonstrated that peripheral nerve (sciatic) lesions trigger a change in caveolae-associated proteins and, as a consequence, transient permeability of the blood–spinal cord barrier (BSCB)^27^. In order to understand if these markers play a central role in the transmittance of cellular signals from remote sites of the brain such as the optic nerve, resulting in alteration in the brain microenvironment during the early phase of BBB compromise, we monitored the changes in tight junctions and activation of microglia/macrophages at 1 day after partial optic nerve transection using immunohistochemical detection of caveolin-1 (Cav-1) and Iba1/ED1 respectively^28^,^29^. Microglia are key mediators of inflammatory responses in the CNS^30^. Importantly, ramified resting microglia respond to subtle changes in the microenvironment arising from an injury resulting in what is commonly referred to as microglial activation. This is accompanied by a graded spectrum of morphological changes that transform ramified resting microglia into activated amoeboid-phagocytic microglia^31^. In the present case, we observed a higher occurrence of ED1 positive activated microglia/macrophages co-localised with Iba1 positive microglia/macrophages not only at the site of injury in the ipsilateral optic nerve but also in the brain at the optic chiasm and the cerebral ventricle when compared to the contralateral optic nerve and control brain (Fig. 2a,e,f). It is also widely recognized that changes in Cav-1, which is a major structural protein of caveolae complexes involved in the transcytosis of numerous substrates including immune mediators, is associated with BBB permeability and consequently matrix metalloproteinases (MMPs) activity in the brain^28^. Herein, we observed a distinct disruption of the tight junction using Cav-1 staining in the cerebral ventricular system at 1 day after partial optic nerve transection, while this disruption was not evident in the control brain (Fig. 2b–d). This disruption in tight junctions at 1 day after injury indicates that EB leakage seen in the brain at 1 day (Fig. 1g) occurs due to BBB disruption rather than axonal uptake and transport of EB from the injury site in the optic nerve to the brain.

**Figure 3:**
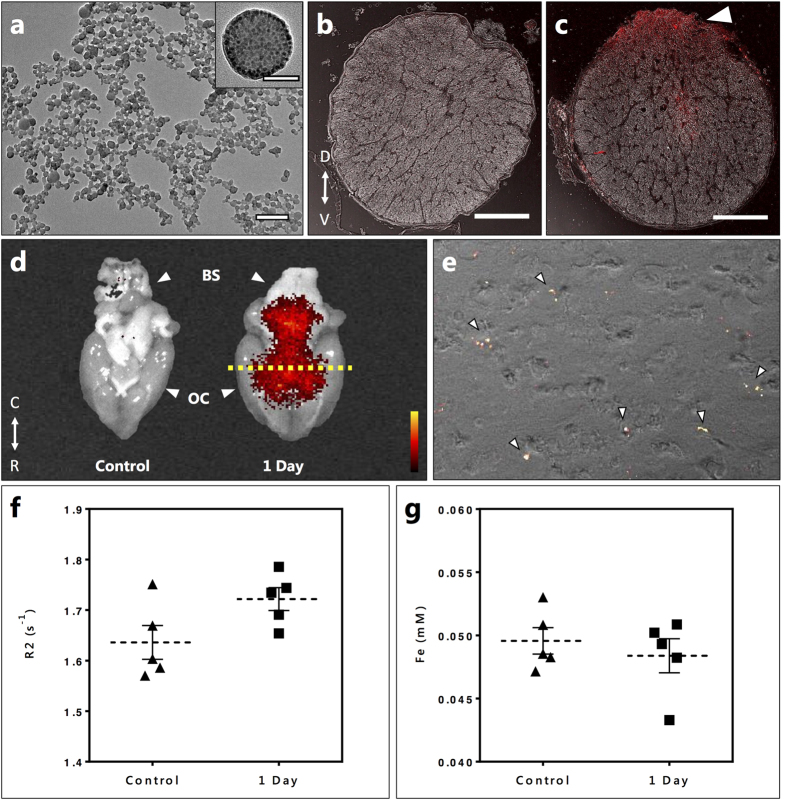
Albumin coated nanoparticles in the brain following partial optic nerve transection. (**a**) TEM image of PGMA-P10-nanoparticles, Inset: High resolution TEM showing magnetite encapsulated in the nanoparticle. Scale Bar: 500 nm, Inset: 50 nm. Confocal images of the transverse section of the (**b**) contralateral (uninjured left optic nerve) and (**c**) ipsilateral (injured right optic nerve) with visible P10 florescence (red) at the injury site from the albumin-coated nanoparticles administered *via* tail vein at 1 day after partial transection. Injury site depicted using white arrow in (**c**). (**d**) Whole-brain multispectral fluorescence images showing: (left, control) no fluorescence signal from an uninjured brain and (right, 1 day) intense red fluorescence signal of P10 dye at the ventral side of the brain from the nanoparticles administered *via* tail vein at 1 day after partial transection. Brain Stem (BS) and optic chiasm (OC) are indicated with white arrows. Yellow dashed line indicates region of brain tissue sectioned for (**e**). (**e**) Transmitted light image with overlaid confocal microscopy image of brain tissue indicating the presence of tail vein injected albumin-functionalised nanoparticles in the brain parenchyma at 1 day after partial transection. Nanoparticles indicated by white arrows. (**f**) Proton transverse relaxation rate: R2 (two-tailed unpaired t-test, P = 0.0601, n = 5 per group) and (**g**) iron concentration in (mM) (two-tailed unpaired t-test, P = 0.5096, n = 5 per group) from the brain homogenates of control (uninjured) and 1 day (injured) animals following nanoparticle administration *via* tail vein.

Having established that secondary degeneration initiated in the optic nerve triggers a remote transient dysfunction of the BBB, we next analyzed whether this remote opening alters the bio-distribution of albumin-coated nanoparticles injected intravenously. Herein, 6-maleimidohexanoic acid-modified poly(glycidyl methacrylate) (PGMA-MAL) was used to produce polymeric nanoparticles. Maleimide functionalization was chosen for its high specificity to thiols, and in particular because of its ability to bind to the free thiol group at cysteine-34 of albumin^32^. Importantly, maleimide-albumin chemistry is a proven methodology to enhance the bioavailability of drugs for intravenous administration. The PGMA-MAL nanoparticles were functionalized with bovine serum albumin (BSA), resulting in 0.11 mg of albumin bound per mg of PGMA-MAL nanoparticles. Additionally, the polymer nanoparticles were encapsulated with magnetite (Fe_3_O_4_) nanoparticles and a highly emissive far-red/near-infrared fluorescent conjugated dye, poly[(9,9-dihexylfluorene)-co-2,1,3-benzothiadiazole-co-4,7-di(thiophen-2-yl)-2,1,3-benzothiadiazole] (P10)^33^, rendering these constructs multimodal, i.e. enabling biodistribution evaluation by magnetic resonance relaxometry (MRR) and fluorescence imaging (Fig. 3 and Supplementary Figs 3d and 5). The albumin-coated PGMA-MAL nanoparticles had a Z-average hydrodynamic diameter of 190 nm (PDI: 0.148) with a zeta potential of −27 mV and a transverse relaxivity (r2) of 234 s^−1^ mM^−1^ Fe at 1.4 T (Supplementary Fig. 3b,c). The toxicity of the nanoparticles was initially examined in rat pheochromocytoma neural progenitor (PC12) cells after 24 h incubation. There was no decrease in cell viability (p < 0.05) for any of the tested concentrations (up to 250 μg ml^−1^) (Supplementary Fig. 4). Nanoparticle suspensions (10 mg/kg dose) were administered by tail vein injection at 1 day following partial optic nerve transection to monitor their passage and consequent biodistribution into the CNS at the early stages of BBB dysfunction. Tissues were harvested from perfused injured (1 day) and control rats 4 hours after intravenous nanoparticle administration. Analysis of optic nerve sections by confocal microscopy indicated the presence of nanoparticles in the ipsilateral optic nerve reflected by intense P10 fluorescence, which was absent in the contralateral optic nerve (Fig. 3b,c). These results matched our EB extravasation studies, indicating that the albumin-coated nanoparticles access the CNS *via* the breached BBB. Furthermore, confocal microscopy and multispectral imaging of sectioned and whole brains respectively indicated the presence of nanoparticles in the brain of injured rats, reflected by intense P10 fluorescence which was absent in the brains of uninjured controls (Fig. 3d,e). It should be noted that the spectral profile and detection sensitivities of both EB and P10 fluorescence signals are different. Even though this prevents exact spatial comparison between EB and P10 signals it nevertheless confirms the presence of the nanoparticles in the brain.

Our results were further substantiated by MRR and ICP-AES measurements of homogenized rat brains. Superparamagnetic Fe_3_O_4_ nanoparticles are effective contrast agents for magnetic resonance (MR) imaging as they possess a large magnetic moment and are free to align with an applied magnetic field. The rate of decay of spin echo recoverable proton transverse magnetisation (R2) within tissues was used as a surrogate indicator of nanoparticle concentration as the presence of magnetite nanoparticles in organs is expected to elevate tissue R2 values disproportionately above that of naturally occurring tissue iron deposits. In the present case relaxometry measurements indicated a trend towards a higher mean R2 in the brains at 1 day following partial optic nerve transection compared to the controls (Fig. 3f), despite similar mean total iron concentrations by ICP-AES analysis (Fig. 3g). The relaxometry results suggest there was a small increase in the concentration of nanoparticles in the brains of the injured rats compared to the controls, supporting the confocal microscopy and multispectral images (Fig. 3d,e), indicating that the intravenously administered albumin-coated PGMA-MAL nanoparticles accumulated in the brain as a consequence of a remote injury in the CNS. However, the greater accumulation of nanoparticles in the injured animals appeared insufficient to be detected by total Iron ICP-AES measurements. It is noteworthy that biodistribution analysis in organs besides the brain by MRR and ICP-AES indicated a large hepatic and splenic accumulation of the nanoparticles in both injured and control animals, with no significant difference between the two (Supplementary Fig. 5).

In summary, the BBB plays the critically important role of maintaining brain homeostasis by ensuring a highly restrictive barrier. Currently, nanoparticle biodistribution, toxicity and safety evaluations are limited to models where the status of the BBB remains unchecked. In this study, we have demonstrated for the first time that remote injury in the CNS (optic nerve) can cause a transient opening of the BBB in the brain which enables intravenously administered albumin-coated nanoparticles to enter the brain in addition to the primary injury site in the optic nerve. These findings provide an important insight to understand possible reasons for BBB accumulation of large nanoparticles. More importantly while our study demonstrates that the propagation of secondary degeneration from the primary injury site and concomitant BBB dysfunction is associated with activation of microglia/macrophages, it is of utmost importance to note that secondary degeneration is a multifactorial condition attributed to a variety of mechanisms evolving from primary cellular damage, including inflammation, oxidative stress, loss of calcium homeostasis and vascular change^34^,^35^,^36^. Indeed it is now widely accepted that an effective approach for attenuating the spread of secondary degeneration will involve multiple neuroprotective therapies administered simultaneously before the many neurotoxic triggers are initiated and become irreversible^37^. Importantly, this study shows that there exists a therapeutic window of opportunity as a result of transient BBB changes in the adult brain for the intravenous administration of multiple neuropharmaceutics using nanotechnology to prevent the progression of secondary degeneration as a consequence of injury in the CNS.

## Methods

### Animals

Adult female PVG hooded rats (160–180 g, ~3 months) were used. Procedures conformed to “Principles of laboratory animal care” (NIH publication No. 86–23, revised 1985) and were approved by the University of Western Australia’s Animal Ethics Committee.

### Partial optic nerve transection

The procedure was similar to that described previously^17^,^18^. Briefly, following anaesthesia the skin overlying the skull was incised along the midline, retracted, and the right optic nerve accessed by deflecting the Harderian lachrymal gland immediately behind the right eye. The nerve parenchyma was exposed about 1 mm behind the eye by making a slit in the dura mater. A controlled 200 μm cut (~1/4 of the optic nerve width) was made in the dorsum of the optic nerve. Care was taken not to stretch the optic nerve or damage major ophthalmic blood vessels. The deflected tissue was replaced and the skin was sutured.

### Evans Blue administration

Adult rats were randomized into 1 day, 3 days, 1 week following partial optic nerve transection and control groups. All groups except the control had partial transection of the right optic nerve with survival for 0, 1 day, 3 days and 1 week. EB was administered intravenously at 1 day, 3 days and 1 week after injury. *via* tail vein injection of a 2% EB dye solution (2.8 ml/kg of 2% w/v dye in sterile saline) which was allowed to circulate for 1 hour before euthanasia and perfusion. The left and right optic nerves and brains were harvested and evaluated for BBB dysfunction using confocal microscopy (1 day, 3 days post injury and control), whole brain multispectral imaging (1 day post injury, control and normal) and quantitative spectrofluorimetric analysis (n = 5 per group; 1 day, 3 days, 1 week post injury and control).

### Nanoparticle synthesis, characterisation and administration

Nanoparticles were prepared using a nonspontaneous emulsification route. Briefly, the organic phase was prepared by dispersing magnetite nanoparticles (5 mg) and dissolving PGMA-MAL (100 mg) and P10 dye (5 mg) in a 1:1:2 mixture of CHCl_3_:THF:MEK (1.5 ml:1.5 ml:3 ml). This organic phase was added drop-wise to a rapidly stirring aqueous solution of Pluronic^®^ F-108 (12.5 mg/ml, 30 ml) and the emulsion was homogenised with a 20 kHz probe-type ultrasonicator at 4W_rms_ for 1 min. The organic solvents were evaporated under reduced pressure at 40 °C. Centrifugation at 3000 g for 45 min removed large aggregates of magnetite, excess polymer and excess P10 dye. The supernatant containing the nanoparticles was further purified using a magnetic separation column. Albumin was covalently attached to PGMA-MAL nanoparticles by reacting the free thiol group at cysteine 34 of albumin with the maleimide double bond to form a thioether linkage under stirring at 37 °C overnight. The nanoparticles were then characterised by dynamic light scattering (DLS), zeta potential measurements, transmission electron microscopy and albumin binding assay. For both the 1 day and control groups, albumin-coated PGMA-Mal nanoparticles suspended in saline was administered intravenously to rats *via* tail vein injection (10 mg/kg) and allowed to circulate for 4 h before euthanasia and perfusion (note: for the 1 day post injury group the time point of albumin-coated PGMA-MAL nanoparticle administration was 1 day after injury). Biodistribution analysis was performed using confocal imaging (n = 8 per group, 1 day post injury and control), multispectral whole brain imaging (n = 3 per group, 1 day post injury and control), magnetic resonance relaxometry (n = 5 per group, 1 day post injury and control) and ICP-AES analysis (n = 5 per group, 1 day post injury and control).

## Additional Information

**How to cite this article**: Smith, N. M. *et al*. An Unexpected Transient Breakdown of the Blood Brain Barrier Triggers Passage of Large Intravenously Administered Nanoparticles. *Sci. Rep*. **6**, 22595; doi: 10.1038/srep22595 (2016).

## Supplementary Material

Supplementary Information

## References

[b1] Pardirdge W. M. Blood-brain barrier delivery. Drug Discov Today. 12, 54–61 (2007). 17198973 10.1016/j.drudis.2006.10.013

[b2] Nance E. A. . A dense poly(ethylene glycol) coating improves penetration of large polymeric nanoparticles within Brain tissue. Sci. Transl. Med. 4, 149ra119 (2012). 10.1126/scitranslmed.3003594 PMC3718558 22932224

[b3] Koffie R. M. . Nanoparticles Enhance Brain Delivery of Blood–brain Barrier-Impermeable Probes for *in Vivo* Optical and Magnetic Resonance Imaging. Proc. Natl. Acad. Sci. 108 **(46)**, 18837–42 (2011). 22065785 10.1073/pnas.1111405108 PMC3219113

[b4] Kreuter J. . Apolipoprotein-mediated transport of nanoparticle-bound drugs across the blood-brain barrier. J Drug Target. 10 **(4)**, 317–325 (2002). 12164380 10.1080/10611860290031877

[b5] Wiley D. T., Webster P., Gale A. & Davis M. E. Transcytosis and brain uptake of transferrin-containing nanoparticles by tuning avidity to transferrin receptor. Proc. Natl. Acad. Sci. 110 **(21)**, 8662–8667 (2013). 23650374 10.1073/pnas.1307152110 PMC3666717

[b6] Lu W., Wan J., She Z. & Jiang X. Brain delivery property and accelerated blood clearance of cationic albumin conjugated pegylated nanoparticle. J Control Release 118, 38–53 (2007). 17240471 10.1016/j.jconrel.2006.11.015

[b7] Rao K. S., Reddy M. K., Horning J. L. & Labhasetwar V. TAT-conjugated nanoparticles for the CNS delivery of anti-HIV drugs. Biomaterials 29, 4429–4438 (2008). 18760470 10.1016/j.biomaterials.2008.08.004 PMC2570783

[b8] Kratz F. Albumin as a drug carrier: design of prodrugs, drug conjugates and nanoparticles. J Control Release. 132, 71–183 (2008). 10.1016/j.jconrel.2008.05.010 18582981

[b9] Pardirdge W. M. Drug transport across the blood–brain barrier. J. Cereb. Blood Flow Metab. 32, 1959–1972 (2012). 22929442 10.1038/jcbfm.2012.126 PMC3494002

[b10] Weiss N., Miller F., Cazaubon S. & Couraud P. O. The blood-brain barrier in brain homeostasis and neurological diseases. Biochim Biophys Acta. 1788, 842–857 (2009). 19061857 10.1016/j.bbamem.2008.10.022

[b11] Tornheim P. A., Prioleau G. R. & McLaurin R. L. Acute responses to experimental blunt head trauma. Topography of cerebral cortical edema. J. Neurosurg. 60, 473–480 (1984). 6699691 10.3171/jns.1984.60.3.0473

[b12] Wang J., Hamm R. J. & Povlishock J. T. Traumatic axonal injury in the optic nerve: evidence for axonal swelling, disconnection, dieback, and reorganization. J. Neurotrauma. 28, 1185–1198 (2011). 21506725 10.1089/neu.2011.1756 PMC3136743

[b13] Chan P. H., Schmidley J. W., Fishman R. A. & Longar S. M. Brain injury, edema, and vascular permeability changes induced by oxygen-derived free radicals. Neurology. 34, 315–320 (1984). 6546610 10.1212/wnl.34.3.315

[b14] Steinman L. Elaborate interactions between the immune and nervous systems. Nat. Immunol. 5, 575–581 (2004). 15164017 10.1038/ni1078

[b15] Kerschensteiner M., Stadelmann C., Dechant G., Wekerle H. & Hohlfeld R. Neurotrophic cross-talk between the nervous and immune systems: implications for neurological diseases. Ann Neurol. 53, 292–304 (2003). 12601697 10.1002/ana.10446

[b16] Moalem G. . Autoimmune T cells protect neurons from secondary degeneration after central nervous system axotomy. Nat Med. 5, 49–55 (1999). 9883839 10.1038/4734

[b17] Fitzgerald M., Bartlett C. A., Harvey A. R. & Dunlop S. A. Early events of secondary degeneration after partial optic nerve transection: an immunohistochemical study. J Neurotrauma. 27, 439–452 (2010). 19852581 10.1089/neu.2009.1112

[b18] Fitzgerald M. . Secondary retinal ganglion cell death and the neuroprotective effects of the calcium channel blocker lomerizine. Invest Ophthalmol Vis Sci. 50, 5456–5462 (2009). 19474405 10.1167/iovs.09-3717

[b19] Tennant M. & Beazley L. D. A breakdown of the blood-brain barrier is associated with optic nerve regeneration in the frog. Visual Neuroscience. 9, 149–155 (1992). 1504024 10.1017/s0952523800009615

[b20] Vargas M. E. & Barres B. A. Why Is Wallerian Degeneration in the CNS So Slow? Annu. Rev. Neurosci. 30, 153–179 (2007). 17506644 10.1146/annurev.neuro.30.051606.094354

[b21] Nahirnay P. C., Reeson P. & Brown C. E. Ultrastructural analysis of blood-brain barrier breakdown in the peri-infarct zone in young adult and aged mice. J. Cereb. Blood Flow Metab. 36, 413–425 (2016). 26661190 10.1177/0271678X15608396 PMC4759675

[b22] Mollgard K. & Saunders N. R. The development of the human blood–brain and blood–CSF barriers. Neuropathol. Appl. Neurobiol. 12, 337–358 (1986). 3534622 10.1111/j.1365-2990.1986.tb00146.x

[b23] Butt A. M., Jones H. C. & Abbott N. J. Electrical resistance across the blood–brain barrier in anaesthetized rats: a developmental study. J. Physiol. (Lond) 429, 47–62 (1990). 2277354 10.1113/jphysiol.1990.sp018243 PMC1181686

[b24] Del Valle J., Camins A., Pallàs M., Vilaplana J. & Pelegrí C. A new method for determining blood–brain barrier integrity based on intracardiac perfusion of an Evans Blue–Hoechst cocktail. J. Neurosci. Methods. 174, 42–49 (2008). 18647619 10.1016/j.jneumeth.2008.06.025

[b25] Laterra J., Keep R., Betz L. A. & Goldstein G. W. Blood-Brain Barrier In Siegel G. J., Agranoff B. W., Albers R. W. (eds), Basic Neurochemistry: Molecular, Cellular and Medical Aspects, 6th edition. Philadelphia: Lippincott-Raven (1999).

[b26] Ballabh P., Braun A. & Nedergaard M. The blood-brain barrier: an overview: structure, regulation, and clinical implications. Neurobiol Dis. 16, 1–13 (2003). 15207256 10.1016/j.nbd.2003.12.016

[b27] Beggs S., Liu X. J., Kwan C. & Salter M. W. Peripheral nerve injury and TRPV1-expressing primary afferent C-fibers cause opening of the blood-brain barrier. Mol Pain. 6, doi: 10.1186/1744-8069-6-74 (2010). PMC2984489 21044346

[b28] Liu J., Jin. X., Liu K. J. & Liu W. Matrix metalloproteinase-2-mediated occludin degradation and caveolin-1-mediated claudin-5 redistribution contribute to blood-brain barrier damage in early ischemic stroke stage. J Neurosci. 32, 3044–3057 (2012). 22378877 10.1523/JNEUROSCI.6409-11.2012 PMC3339570

[b29] Ito D. . Microglia-specific localisation of a novel calcium binding protein, Iba1. Brain Res Mol Brain Res. 57, 1–9 (1998). 9630473 10.1016/s0169-328x(98)00040-0

[b30] Nimmerjahn A., Kirchhoff F. & Helmchen F. Resting microglial cells are highly dynamic surveillants of brain parenchyma *in vivo*. Science. 308, 1314–1318 (2005). 15831717 10.1126/science.1110647

[b31] Hains B. C. & Waxman S. G. Activated microglia contribute to the maintenance of chronic pain after spinal cord injury. J Neurosci. 26, 4308–4317 (2006). 16624951 10.1523/JNEUROSCI.0003-06.2006 PMC6674010

[b32] Lu W. . Cationic albumin-conjugated pegylated nanoparticles as novel drug carrier for brain delivery. J Control Release. 107, 428–448 (2005). 16176844 10.1016/j.jconrel.2005.03.027

[b33] Ding D. . Bright far-red/near-infrared conjugated polymer nanoparticles for *in vivo* bioimaging. Small. 9, 3093–3102 (2013). 23625815 10.1002/smll.201300171

[b34] O’Hare Doig R.L., Bartlett C.A., Maghzal G.J., Lam M., Archer M., Stocker R. & Fitzgerald M. Reactive species and oxidative stress in optic nerve vulnerable to secondary degeneration. Exp Neurol 261, 136–146 (2104). 24931225 10.1016/j.expneurol.2014.06.007

[b35] Savigni D. L., O’Hare Doig R. L., Szymanski C. R., Bartlett C. A., Lozić I., Smith N. M. & Fitzgerald M. Three Ca2+ channel inhibitors in combination reduce chronic secondary degeneration following neurotrauma. Neuropharmacology 75, 380–390 (2013). 23958451 10.1016/j.neuropharm.2013.07.034

[b36] Lozić I., Bartlett C. A., Shaw J. A., Iyer K. S., Dunlop S. A., Kilburn M. R. & Fitzgerald M. Changes in subtypes of Ca microdomains following partial injury to the central nervous system. Metallomics 6, 455–64 (2014). 24425149 10.1039/c3mt00336a

[b37] Kuffler D. P. Maximizing neuroprotection: where do we stand? Ther. Clin. Risk Manag. 8, 185–194 (2012). 22547938 10.2147/TCRM.S16196 PMC3333458

