# Supplementary material for: An Unexpected Transient Breakdown of the Blood Brain Barrier Triggers Passage of Large Intravenously Administered Nanoparticles
Source: Sci Rep. 2016 Mar 4;6:22595. doi: 10.1038/srep22595 (PMC4778073; doi:10.1038/srep22595)
Supplement: Supplementary Information [file srep22595-s1.doc]

**Supplementary Information**

**Materials.** All chemicals were purchased from Sigma-Aldrich unless otherwise stated: benzyl ether (99%), iron(III) acetylacetonate (97%), oleic acid (BDH, 92%), oleyl amine (70%), Pluronic® F-108, 1,2-tetradecanediol (90%), 6-maleimidohexanoic acid (90%), bovine serum albumin (lyophilized, 96%), Evans Blue (≥75%) and trichloroacetic acid (>99%) were used as received. All tissue culture reagents were purchased from Invitrogen unless otherwise stated: T75 flasks, bovine serum albumin (Aldrich), fetal bovine serum, L-glutamine 200 mM, GlutaMAX 100, horse serum, MEM, nonessential amino acids (NEAA) 100, penicillin/streptomycin, poly(L-lysine) (Aldrich), RPMI1640, sodium pyruvate 100 , and trypsin/EDTA. Samples were mounted for light microscopy using Fluoromount-G (Southern Biotech). GentleMACS^TM^ M tubes were used to homogenize tissues (Miltenyi Biotec).

Animals

3 months old female PVG hooded rats (160–180 g) were bred at the Animal Resources Centre (Murdoch, WA, Australia), housed under a standard 12 hour light/dark cycle and fed standard rat chow and water ad libitum. Procedures conformed to “Principles of laboratory animal care” (NIH publication No. 86–23, revised 1985) and were approved by The University of Western Australia's Animal Ethics Committee. Rats were anaesthetized with xylazine (llium xylazil, Troy Laboratories, i.p. 10 mg/kg) in combination with ketamine (Ketamil, Troy Laboratories, 50 mg/Kg) and euthanased with Euthal (Pentobarbitone sodium 850 mg/kg; Phenytoin sodium 125 mg/kg; Virbac, i.p.).

Partial optic nerve transection

Briefly, anaesthetized rats were shaved along the midline of the skull towards the right eye and the shaved area was triple swabbed with betadine and 70% ethanol. The skin overlying the skull was incised along the midline, retracted, and the right optic nerve was accessed by deflecting the Harderian lachrymal gland immediately behind the right eye. The nerve parenchyma was exposed about 1 mm behind the eye by making a slit in the dura mater with ophthalmic scissors. A controlled 200 μm cut (~1/4 of the optic nerve width) was made in the dorsum of the optic nerve using a diamond radial keratotomy knife, with the depth determined by the protrusion of the blade beyond a surrounding guard. Care was taken not to stretch the optic nerve or damage major ophthalmic blood vessels. The surround tissue was replaced and the skin was sutured with 4/0 non-absorbable silk suture. Eyes were lubricated with Luxyal Eye drops and rats were placed on a warming blanket for recovery with subcutaneous injections of analgesic (Carprieve, 5 mg kg^-1^ in sterile PBS, Norbrook) and sterile PBS (1 ml). No postoperative infections were observed.

**Evans Blue administration**

Rats were randomized into 1 day, 3 days, 7 days (1 week) and control groups. 1 day, 3 days and 1 week groups had partial transection of the right optic nerve, while the control group consisted of uninjured rats. As sham injury has previously been demonstrated to be not different to control, shams were not included in this study**.**^1^ For 1 day, 3 days and 1 week groups Evans Blue (EB) was administered 1 day, 3 days and 1 week after injury respectively. For all groups, EB dye solution (2.8 ml/kg of 2% w/v dye in sterile saline) was intravenously administered to rats via tail vein injection and allowed to circulate for 1 hour before euthanizing.

***In vivo* qualitative evaluation of Blood Brain Barrier (BBB) integrity**

Qualitative evaluation of vascular permeability in the optic nerves and brains was conducted using two optical imaging techniques to map regions of Evans Blue (EB) fluorescence: (1) confocal microscopy of cryosectioned tissues and (2) whole brain multispectral imaging on a Maestro animal imager (CRi).

For confocal microscopy three groups of rats: 1 day, 3 days and control were used. Following EB administration and euthanasia rats were transcardially perfused-fixed through the left ventricle with non-heparinized saline (0.9% w/v NaCl, 100 ml) to remove the intravascularly localized dye followed by 4 % paraformaldehyde (4% w/v PFA in 0.1 M phosphate buffer pH 7.2, 100 ml). The left and right optic nerves were subsequently harvested, post-fixed in 4 % PFA overnight and cryoprotected by immersion in 15 % sucrose (15% w/v sucrose in PBS) for 24 h at 4°C. The left and right optic nerves were embedded in optimal cutting temperature compound (OCT) and cryosectioned transversely, 14 µm thick sections, before mounting on microscope slides. The reproducibility of the thickness of the sections generated by the cryostat was approximately 2–6%. The sections were imaged under a confocal microscope (Leica TCS SP2 multiphoton confocal microscope) with a 633 nm laser excitation and 680 nm emission (bandwidth 10 nm) (Fig. S1).

For whole brain multispectral imaging three groups of rats: 1 day, control and normal were used. The normal group consisted of uninjured rats that were not administered EB. Following EB dye administration (1 day and control groups) and euthanasia rats were transcardially perfused with non-heparinized saline and freshly harvested whole brains were subsequently imaged using the CRi Maestro 2 multispectral imaging system. To visualise EB fluorescence of whole brains, the green filter was set to a wavelength of 680 nm, with 212.21 ms exposure time. Subsequently, the same brains were embedded in agarose gel and cut coronally into nine 2 mm thick sections from rostral to caudal using a Vibratome. The sections were imaged with the green filter set to a wavelength of 680 nm, with 170 ms exposure time (Fig. S2).

.

For the immunohistochemical analysis, animals were transcardially perfused-fixed with 0.9% saline followed by 4% PFA in 0.1M phosphate buffer pH 7.2, after which the brain and both optic nerves (injured and uninjured) were harvested. Tissues were frozen in OCT compound and cryosectioned at -20^o^C. Transverse brain sections (20µm) were collected at 1mm intervals throughout the brain between the optic chiasm and cerebellum; longitudinal nerve sections (14µm) were collected through the injury site to the optic chiasm. Microglia were identified using goat anti Iba-1 (1:1000 Abcam Ab5076) recognising all microglia/macrophages and mouse anti-ED1 (1:1000 Millipore MAB1435) recognising activated microglia/macrophages. Tight junction proteins were identified using rabbit anti-Caveolin-1 (1:500 Abcam Ab2910). Images were collected on the Nikon A1Si confocal microscope.

***In vivo* quantitative evaluation of Blood Brain Barrier (BBB) integrity**

Four groups of rats: 1 day, 3 days, 1 week and control with n=5 animals per group were used. Following EB administration and euthanasia, blood (0.2 ml) was collected from the heart and rats were transcardially perfused with non-heparinized saline. Freshly harvested whole brains were subsequently immersed in PBS to wash externally localised dye, blotted dry with a paper towel and weighed. To extract the EB dye the brains were homogenized in trichloroacetic acid (50% w/v TCA, volume added was 2 times the tissue weight) in GentleMACS^TM^ M tubes using a Gentle MACS Dissociator (Miltenyi Biotec). The homogenates were centrifuged (10,000 rpm, 20 min), the supernatants were collected, freeze-dried and resuspended in MilliQ water (250 µl). Subsequently, the samples were diluted fourfold with absolute ethanol and EB fluorescence intensity was measured using a multimode plate reader (EnSpire 2300; PerkinElmer) with an excitation wavelength of 620 nm (bandwidth 10 nm) and an emission wavelength of 680 nm (bandwidth 10 nm). Calculations were based on external EB standards in the same solvent (0.1 – 6.45 μg/ml) and the concentration of EB was expressed as µg/g of tissue. The EB content of plasma was similarly determined where blood samples were centrifuged (10,000 rpm, 10 min), the supernatants were collected and diluted 1:3 with 50% TCA (w/v) and centrifuged again (10,000 rpm, 10 min). The resulting supernatants were diluted fourfold with absolute ethanol and EB fluorescence intensity was measured. The amount of EB in the brain was expressed as percentage of EB injected by determining the ratio of brain to plasma EB content.

For the EB standard curve, a 3% EB dye solution (3% w/v EB dye in 50% w/v TCA, 5 mL) was diluted 1:1000 with 50 % TCA:Ethanol (1:3). The resulting EB stock solution (30 µg/ml) was further diluted with 50% TCA:Ethanol (1:3) to obtain a standard solution at 6.45 µg/mL for the first point of the standard curve. A 2x dilution series was then performed to obtain standard solutions at 3.225, 1.6125, 0.806, 0.403, 0.2015, and 0.1μg/ml. EB fluorescence in these samples was measured (4 replicates for each concentration) using a multimode plate reader (EnSpire 2300) with an excitation wavelength of 620 nm (bandwidth 10 nm) and an emission wavelength of 680 nm (bandwidth 10 nm).

**Synthesis of maleimide-modified PGMA:** PGMA was synthesized by radical polymerization according to a previously published procedure.^2^ Briefly, glycidyl methacrylate was polymerized in methyl ethyl ketone (MEK) to give PGMA (Mw = 250 000 g/mol), using azobisisobutyronitrile as initiator. The polymer was purified by multiple precipitations from MEK solution using diethyl ether. To attach maleimide to the polymer, a solution of 6-maleimidohexanoic acid (147.8 mg) and PGMA (100 mg) in MEK (30 ml) was heated at 70°C under a flow of N_2 (g)_ for 4 h with moderate stirring (500-700 rpm) (Fig. S3). The solution was reduced *in vacuo* at 40 °C (final volume ~1-2 ml) before the modified polymer (PGMA-MAL) was precipitated by drop-wise addition to diethyl ether (20 ml). The ether was decanted, the modified polymer was air dried and stored at room temperature for characterization and subsequent use. **^1^HNMR:** δ_H_ (500 MHz, CDCl_3_) 6.68 (s, maleimide olefin), 4.29 (br s, PGMA), 3.81 (br s, PGMA), 3.47 (m, maleimide), 3.22 (br s, PGMA), 2.83 (br s, PGMA), 2.62 (br s, PGMA), 2.33 (m, maleimide), 1.89 (br m, PGMA), 1.64 (m, maleimide), 1.61 (m, maleimide) 1.21 (m, maleimide), 1.00 (br m, PGMA).

**Preparation of magnetite nanoparticles.** Magnetite (Fe_3_O_4_) nanoparticles were synthesized by the organic decomposition of Fe(acac)_3_ in benzyl ether at 300^o^C, in the presence of oleic acid, oleylamine, and 1,2-tetradecanediol, as previously described by Sun *et al*.^3^

**Preparation of P10 dye.** P10 dye was synthesized as previously described Ding *et al*.^4^

**PGMA-MAL nanoparticle synthesis.** Nanoparticles were prepared using a nonspontaneous emulsification route. The organic phase was prepared by dispersing magnetite nanoparticles (5 mg) and dissolving PGMA-MAL (100 mg) and P10 dye (5 mg) in a 1:1:2 mixture of CHCl_3_:THF:MEK (1.5 ml:1.5 ml:3 ml). This organic phase was added dropwise to a rapidly stirring aqueous solution of Pluronic® F-108 (12.5 mg/ml, 30 ml) and the emulsion was homogenised with a 20 kHz probe-type ultrasonicator at 4W_rms_. The organic solvents were evaporated under reduced pressure at 40 °C. Centrifugation at 3000g for 45 min removed large aggregates of magnetite, excess polymer and excess P10 dye. The supernatant was loaded onto a magnetic separation column (LS, Miltenyi Biotec) in the presence of a rare earth magnet and nanoparticles without magnetite were washed through the column with Pluronic solution (2 x 5 mL). To elute the magnetic nanoparticles, the magnet was removed and the column was washed with Pluronic solution (~1-2 mL). The resulting concentrated suspension of PGMA-MAL nanoparticles encapsulating magnetite and P10 dye was aliquoted, covered with foil and stored at 4°C for quantification by lyophilization, characterization and subsequent use.

**Conjugation of albumin to PGMA-MAL nanoparticles.** Albumin was covalently attached to PGMA-MAL nanoparticles by reacting the free thiol group at cysteine 34 of albumin with the maleimide double bond to form a thioether linkage. In brief, a suspension of PGMA-MAL nanoparticles (5 mg/ml in Pluronic® solution) was centrifuged at 24000g for 20 min, the supernatant was decanted and replaced with a BSA solution (50mg/ml in Milli-Q water). The nanoparticles were resuspended in the BSA solution with a probe-type ultrasonicator at low power for 1 min and incubated at 37°C in an orbital incubator under constant stirring/shaking overnight. The resulting suspension of albumin-coated PGMA-MAL nanoparticles encapsulating magnetite and P10 dye was covered with foil and stored at room temperature for characterization and subsequent use. The nanoparticle suspension was sterilized by UV irradiation prior to conducting *in vitro* and *in vivo* experiments.

**Albumin binding assay.** To quantify albumin binding to PGMA-MAL nanoparticles, a suspension of PGMA-MAL nanoparticles (0.5 mg/ml in Milli-Q water, 2 ml) containing BSA (5 mg/ml) was incubated for 18 h at room temperature. The suspension was centrifuged, the supernatant was assayed for BSA at 280 nm on the NanoDrop 2000 spectrophotometer (Thermo Fisher Scientific) and compared to a control solution containing only BSA (5 mg/ml, 2 ml). Albumin binding was determined to be 11% w/w of PGMA-MAL nanoparticles.

**Dynamic light scattering (DLS) and zeta potential measurements.** DLS and zeta potential (ζ) measurements to measure the hydrodynamic diameters and zeta potential of the nanoparticles were performed using a Zetasizer Nano instrument (Malvern). Synthesized polymer nanoparticles were diluted in water at a concentration of 200 µg/ml. All measurements were recorded at 25°C in disposable clear folded capillary cells (Malvern) in triplicate (Fig. S3).

**Transmission Electron Microscopy (TEM).** Synthesized PGMA-MAL polymer nanoparticles were drop-casted on carbon-coated copper TEM grids and imaged at an accelerating voltage of 120 kV on a JEOL 2100 transmission electron microscope.

**Cell culture.** Rat pheochromocytoma (PC12) cells were cultured in poly-(L-lysine)-coated polystyrene flasks in a humidified atmosphere containing 5% CO_2_ at 37°C, and maintained in RPMI1640 medium supplemented with horse serum (10% v/v), fetal bovine serum (5% v/v), L-glutamine (2 mM), penicillin-streptomycin (100 U ml^-1^, 100 µg ml^-1^), non-essential amino acids (100 µM) and sodium pyruvate (1 mM).

**Cell viability measurements.** For cell viability measurements, cells were seeded in 96-well plates coated with poly-(L-lysine) at a cell density of 2 x 10^5^ cells/ml and incubated in a humidified atmosphere containing 5% CO_2_ at 37°C for 24 h. Following this, the cell media was replaced with albumin-coated PGMA-MAL nanoparticle suspensions of different concentrations (0, 1, 10, 100 and 250 µg/ml) in media, where each concentration was added in triplicate. After a further 24 h incubation, the nanoparticle suspension was removed, the cells were washed once with PBS, and 100 μL of Live/Dead reagents was added (calcein AM, 1 μM; ethidium homodimer-1, 2 μM). After 30 min, images were recorded using an inverted fluorescence microscope at 20x magnification (Olympus IX-51). Three images were recorded from each well at consistent locations for all wells. Live and dead cells were counted using Image J analysis software with a cell counter plugin and the cell viability for each nanoparticle concentration was expressed as a percentage of living cells (Fig. S4).

**Confocal microscopy of albumin-coated PGMA-MAL nanoparticles.** For confocal microscopy two groups of rats: 1 day and Control were used. The 1 day group had partial transection of the right optic nerve, while the control group consisted of uninjured rats. For both the 1 day and control groups, albumin-coated PGMA-Mal nanoparticles suspended in Milli-Q water was administered intravenously to rats *via* tail vein injection (10 mg/kg) and allowed to circulate for 4 h before euthanizing (note: for the 1 day group the time point of albumin-coated PGMA-Mal nanoparticle administration was 1 day after injury). Following euthanasia, rats were transcardially perfused-fixed through the left ventricle with non-heparinized saline (0.9% w/v NaCl, 100 ml) followed by 4 % PFA in 0.1 M phosphate buffer pH 7.2 (100 ml). The left and right optic nerves and brains were subsequently harvested, post-fixed in 4 % PFA overnight and cryoprotected by immersion in 15 % sucrose (15% w/v sucrose in PBS) for 24 h at 4 °C. The brains were cut into left and right hemispheres and tissues were embedded in OCT compound and cryosectioned transversely (optic nerves - 14 µm; brain - 20 µm) before mounting on microscope slides. The reproducibility of the thickness of the sections generated by the cryostat was approximately 2–6%. The sections were imaged under a confocal microscope (Leica TCS SP2 multiphoton confocal microscope) with an excitation wavelength of 458 nm (100% power)  and 488 (5% power), the 488 nm laser was used to obtain the phase image, and emission wavelength of 700 nm.

**Whole-brain multispectral fluorescence imaging of albumin-coated PGMA-MAL nanoparticles.** For whole brain Maestro multispectral imaging three groups of rats: 1 day, control and normal were used. The 1 day group had partial transection of the right optic nerve, while the control and normal groups consisted of uninjured rats. For both the 1 day and control groups, albumin-coated PGMA-MAL nanoparticles suspended in Milli-Q water was administered intravenously to rats *via* tail vein injection (10 mg/kg, 500 μl) and allowed to circulate for 4 h before euthanizing (note: for the 1 day group the time point of albumin-coated PGMA-MAL nanoparticles administration was 1 day after injury). For the normal group rats were intravenously administered saline via tail vein injection (500 μl) and were euthanized 4 h post injection. Following euthanasia rats were transcardially perfused with non-heparinized saline and freshly harvested whole brains were subsequently imaged using the CRi Maestro 2 multispectral imaging system. The fluorescence signal of P10 dye encapsulated in the albumin-coated PGMA-MAL nanoparticles was detected with an excitation wavelength of 460 nm and an emission wavelength of 700 nm.

**Relaxometry and Inductively Coupled Plasma Atomic Emission Spectroscopy (ICP-AES).** For relaxometry and ICP-AES two groups of rats: 1 day and control n=5 rats per group were used. The 1 day group had partial transection of the right optic nerve, while the control group consisted of uninjured rats. For both the 1 day and control groups, albumin-coated PGMA-MAL nanoparticles suspended in Milli-Q water was administered intravenously to rats via tail vein injection (10 mg/kg, 500 μL) and allowed to circulate for 4 h before euthanizing (note: for the 1 day group the time point of albumin-coated PGMA-MAL nanoparticles administration was 1 day after injury). Following euthanasia rats were transcardially perfused with non-heparinized saline (0.9% w/v NaCl, 100 ml) and freshly harvested whole brains and reticuloendothelial system (RES) organs (liver, kidneys and spleen) were weighed and homogenised in sterile PBS (PBS volume: 2x organ weight for liver, kidneys and brain and 6x organ weight for spleen). The resulting homogenates were aliquoted (0.5 ml x 5) into relaxometry tubes and incubated in a 37.4 °C water bath. Subsequently, the relaxivity data were measured using a Minispec mq series instrument (Bruker) operating at 1.41 T. A Carl-Purcell-Meiboom-Gill (CPMG) spin echo sequence was used to measure T2. The echo spacing was 0.5 ms for the short TE measurements (liver, kidney and spleen – 2,000 echoes; brain – 3,000 echoes) and 2 ms and for the long TE measurements (liver, kidney and spleen - 500 echoes; brain - 750 echoes). An inversion recovery (IR) sequence was used to measure T1 using 10 inversion times (TI) logarithmically spaced between 50 and 10,000 ms (Fig. S5).

Following relaxivity measurements, all samples were acid digested and the iron content was assessed by ICP-AES (Marine and Freshwater Research Laboratory Environmental Science, Murdoch University). Briefly, samples were transferred to weighed acid digest tubes and sample weights were recorded. Concentrated nitric acid (10 ml) was added and samples were heated at 95 °C for 4-6 h (final volume ~ 1 ml). The digests were then cooled to room temperature and diluted with the addition of Milli-Q water (4 ml). Sample weights were recorded, samples were transferred to 5 ml tubes for ICP-AES analysis (Fig. S5).

**Supporting Information References**

1. Fitzgerald, M. et al. Secondary degeneration of the optic nerve following partial transection: The benefits of lomerizine. *Exp. Neurol.* **216,** 219-230 (2009).
2. Swaminatha Iyer, K., Zdyrko, B., Malz, H, Pionteck, P. & Luzinov, I. Polystyrene layers grafted to macromolecular anchoring layer. *Marcromolecules*. **36**, 6519-6526 (2003).
3. Sun, S. et al. Monodisperse MFe_2_O_4_ (M = Fe, Co, Mn) Nanoparticles. *J. Am. Chem. Soc.* **126**, 273-279 (2004).
4. Ding, D. et al. Bright far-red/near-infrared conjugated polymer nanoparticles for in vivo bioimaging. *Small*. **9**, 3093-3102 (2013).

**Figure S1**


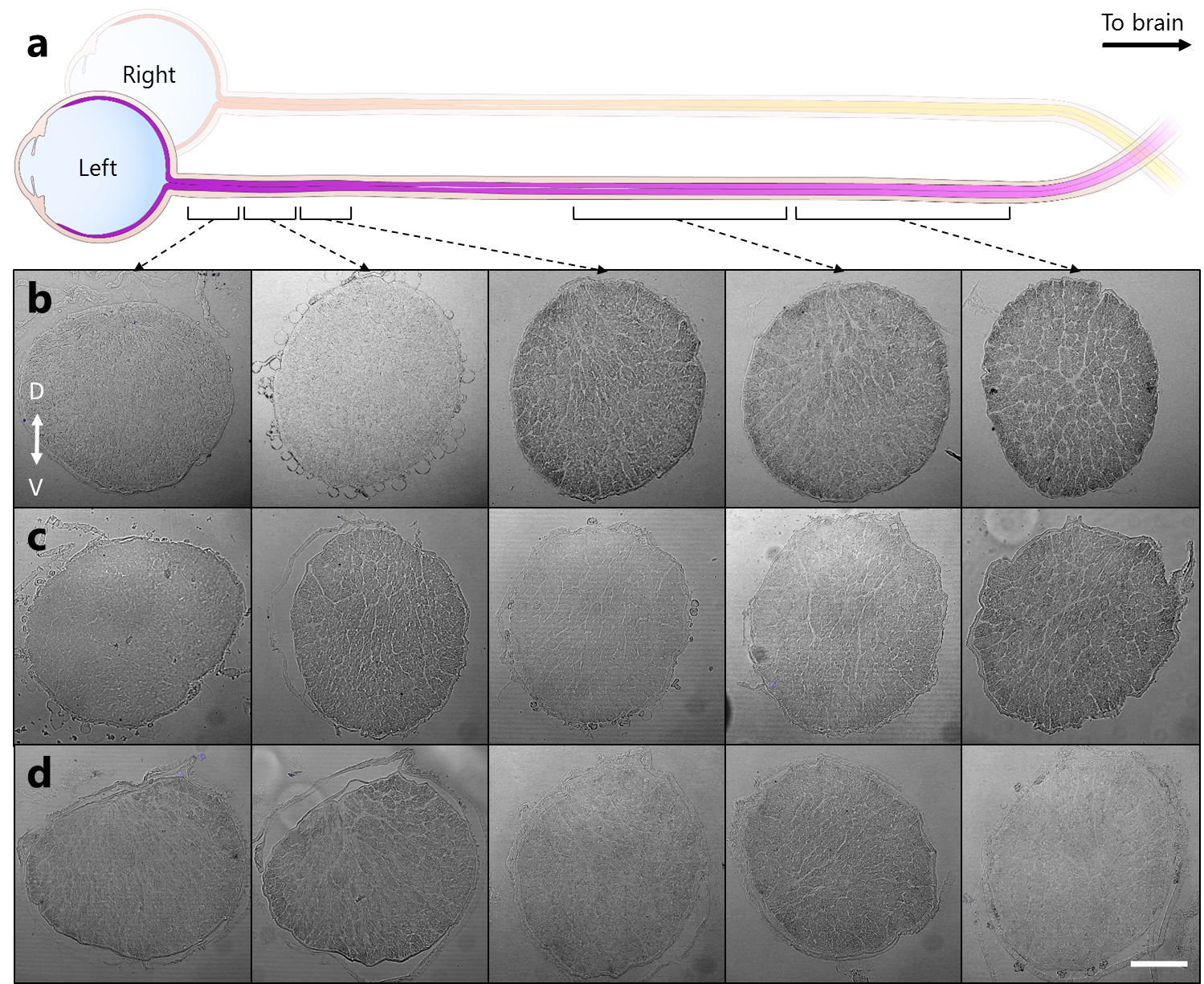


**Figure S1**. **(a)** Schematic of sampled left (uninjured) optic nerve regions. **(b-d)** Transmitted light images with overlaid **c**onfocal EB fluorescence images of transverse sections of the left optic nerve of (**b**) control following EB administration, (**c & d**) injured following EB administration at **(c)** 1 day and **(d)** 3 days after injury. D and V represent dorsal and ventral side of the optic nerve, respectively. The optic nerves are positioned with the dorsal side upwards. Sections are positioned from left to right, based on proximity to eye (left) and the brain (right). **b-d** Scale bar 100 µm.

**Figure S2**

**
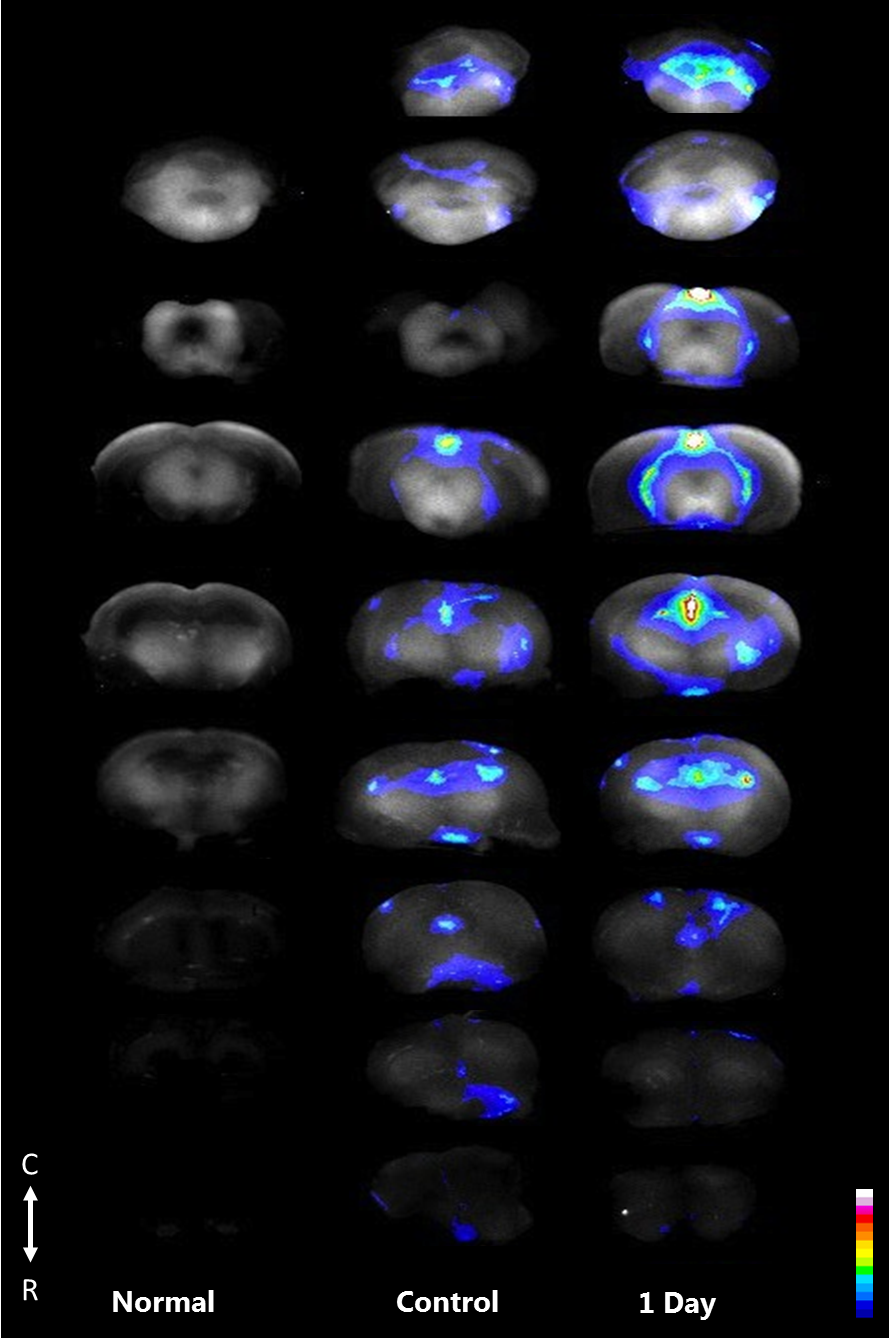
**

**Figure S2**. Multispectral heat maps of EB fluorescence intensity obtained from coronal sections of the whole brain: (left, Normal) uninjured with no EB administration; (middle, control) uninjured with EB tail vein injection; (right, 1 day) injured with EB tail vein injection 1 day after injury. C and R represent caudal and rostral side of the brain, respectively; colour code represents degree of EB vascular leakage, where white/red is high and dark blue is low.

**Figure S3**

**
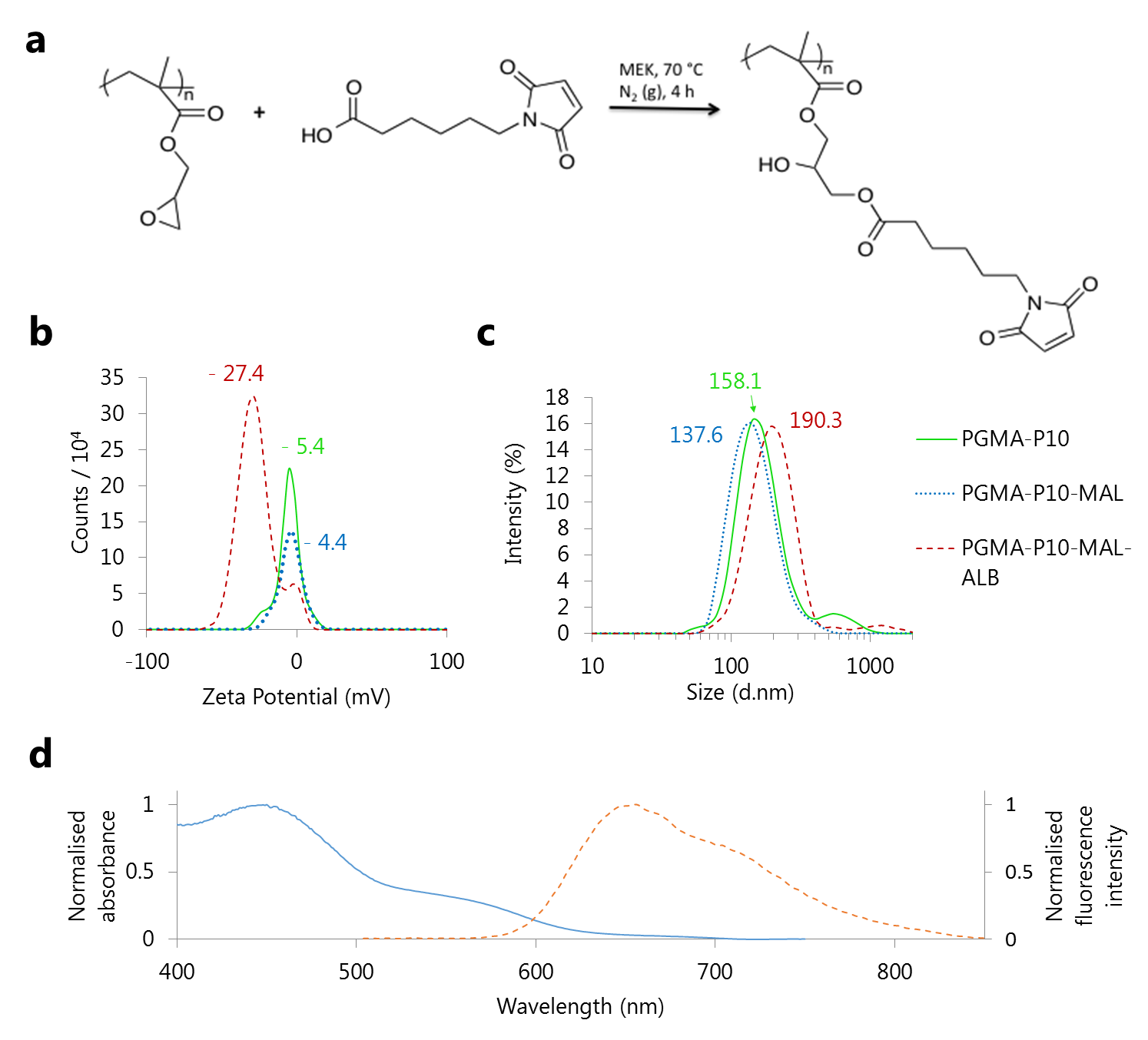
**

**Figure S3**. **(a)** Chemical representation of the synthesis of maleimide-functionalised PGMA from PGMA and 6-maleimidohexanoic acid. **(b)** Zeta potential distributions of PGMA nanoparticles encapsulating P10 and magnetite (green), after maleimide attachment (blue) and after albumin attachment (red). **(c)** Particle size distributions of PGMA nanoparticles encapsulating P10 and magnetite (green), after maleimide attachment (blue) and after albumin attachment (red). **(d)** Spectral characteristics of the P10 dye encapsulated in albumin-coated PGMA-MAL nanoparticles showing normalised absorbance (blue solid line) and normalised fluorescence (red dashed line) at 450 nm excitation.

**Figure S4**


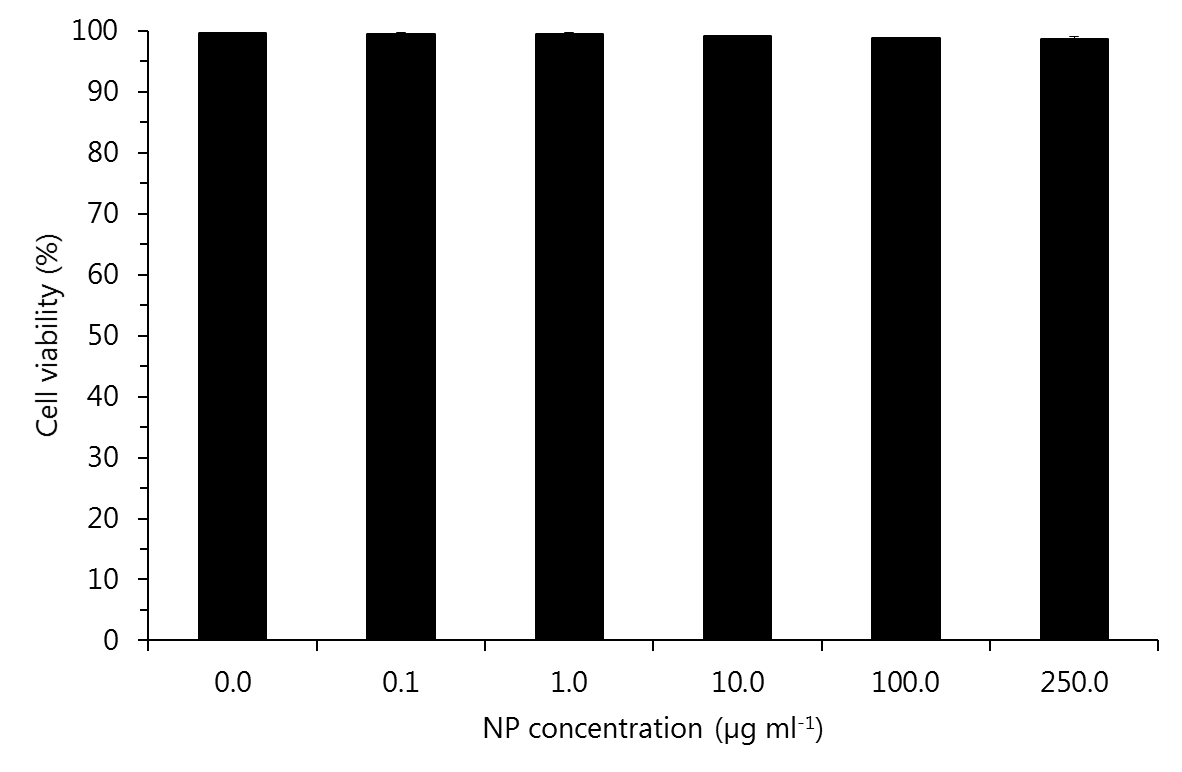


**Figure S4**. Viability of PC12 cells incubated with increasing concentrations of albumin-coated PGMA-MAL nanoparticles. Immortalized PC12 cells incubated with nanoparticles for 24 h displayed no statistically significant (one-way ANOVA with Bonferroni *post-hoc* correction, *p* < 0.05, *n* = 3 per group) reduction in cell viability (mean ± SD) for concentrations up to 250 μg/ml.

**Figure S5**


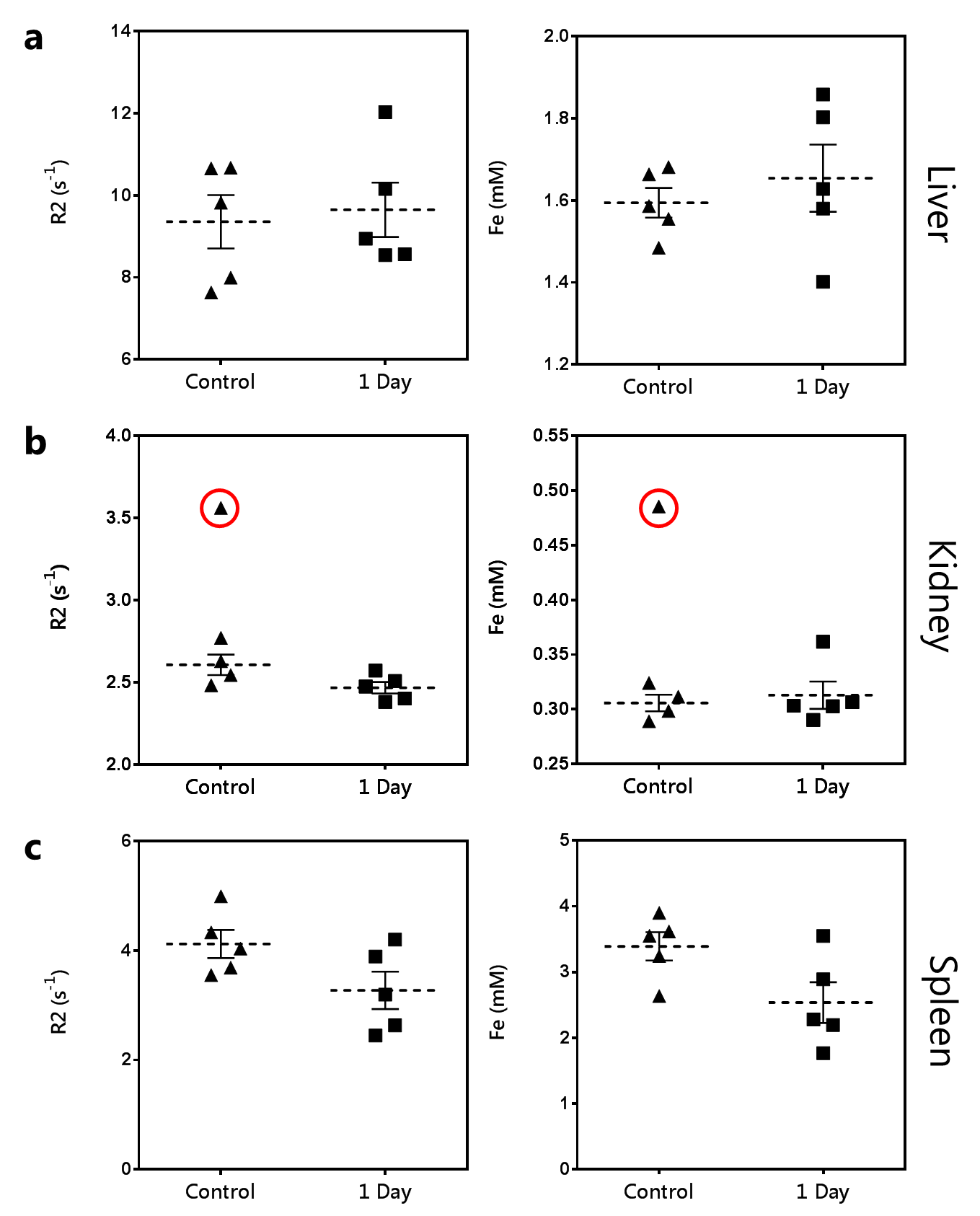


**Figure S5**. Proton transverse relaxation rate: R2 (**a-c**, left) and iron concentration in (mM) (**a-c**, right) from reticuloendothelial system (RES) organ homogenates **(a)**Liver **(b)** Kidney and **(c)** Spleen of control (uninjured) and 1 day (injured) animals following nanoparticle administration *via* tail vein. **(a)** Left: Liver R2 (two-tailed unpaired t-test, P = 0.7616, n=5 per group); Right: Liver iron concentration (two-tailed unpaired t-test, P = 0.5212, n=5 per group) **(b)** Left: Kidney R2 (two-tailed unpaired t-test, P = 0.0787, n=5 per group); Right: Kidney iron concentration (two-tailed unpaired t-test, P = 0.6657, n=5 per group); Outliers (circled in red) identified using Grubbs' test (alpha = 0.05) were excluded from statistical analysis. **(c)** Left: Spleen R2 (two-tailed unpaired t-test, P = 0.0831, n=5 per group); Right: Spleen iron concentration (two-tailed unpaired t-test, P = 0.0536, n=5 per group)
